# SH3-domain binding protein 1 in the tumor microenvironment promotes hepatocellular carcinoma metastasis through WAVE2 pathway

**DOI:** 10.18632/oncotarget.7786

**Published:** 2016-02-28

**Authors:** Yiming Tao, Kuan Hu, Fengbo Tan, Sai Zhang, Ming Zhou, Jia Luo, Zhiming Wang

**Affiliations:** ^1^ Department of Hepatobiliary Surgery, Xiangya Hospital, Central South University, Changsha, Hunan, China; ^2^ Institute of Medical Sciences, Xiangya Hospital, Central South University, Changsha, Hunan, China; ^3^ Institute of Cancer Research, Central South University, Changsha, Hunan, China; ^4^ Department of Hepatobiliary Surgery, Hunan Provincial Tumor Hospital, Changsha, Hunan, China

**Keywords:** hepatocellular carcinoma, SH3-domain binding protein 1, Wiskott-Aldrich syndrome family verproline-homologous protein 2, metastasis, prognosis

## Abstract

SH3-domain binding protein-1 (SH3BP1) specifically inactivating Rac1 and its target WAVE2 is required for cell motility. The present study shows SH3BP1 expression patterns in human HCC tissues and cell lines were examined. The regulation of SH3BP1 on HCC cell migration and invasion related to Rac1-WAVE2 signaling was characterized using *in vitro and in vivo* models. SH3BP1 overexpressed in HCC tissues and highly metastatic HCC cells was significantly associated vascular invasion (VI). SH3BP1 promoted VEGF secretion via Rac1-WAVE2 signaling, so as to exert an augmentation on cell invasion and microvessel formation. In three study cohorts with a total of 516 HCC patients, high SH3BP1 expression combined with high microvessel density (MVD) was confirmed as a powerful independent predictor of HCC prognosis in both training cohorts and validation cohort. Being an important angiogenic factor of HCC through Rac1-WAVE2 signaling, SH3BP1 promotes tumor invasion and microvessel formation contributing to HCC metastasis and recurrence. SH3BP1 is a novel WAVE2 regulator, a prognostic marker and a potential therapeutic target of HCC.

## INTRODUCTION

Hepatocellular carcinoma (HCC) is the sixth most common primary liver cancer and the third greatest cause of death from cancer worldwide [1, 2]. Despite improved diagnostic and treatment strategies, among which hepatic resection is one of the first priorities. The overall survival of patients with HCC remains poor because of a high incidence of recurrence and metastasis [3, 4]. Thus, continued searching for molecular markers, which is helpful to predict and inhibit recurrence and metastasis, is of great importance in HCC therapy.

The recurrence and metastasis of HCC is a multi-step process that often involves many complex biological and pathological events [5, 6]. The accumulated evidence suggests that tumor metastasis is to a large extent attributable to cancer cell migration [7]. Rac1 was reported to stimulate lamellipodium formation and contribute to cancer cell invasion by regulating activation of WAVE2 signaling complex [8-10]. We previously demonstrated that WAVE2 expression was significantly correlated to vein invasion in HCC [11]. Rac-WAVE2 was also reported to be essential for invasion and metastasis of murine melanoma [12]. However, the molecular mechanisms of Rac1-WAVE2-regulated invasion in HCC needed to be further elucidated. As previously documented, HIF-1α is a crucial factor of hypoxia-induced tumor angiogenesis [10, 13]. Rac1 increase VEGF and promoted HCC angiogenesis *via* direct interaction with HIF-1α to gain an increased HIF-1α stability [14, 15].

Recent studies demonstrated that SH3BP1 (SH3-domain binding protein-1, also known as 3BP-1) belonging to RhoGAP family was fundamentally required for cell motility, because it could activated by guanine nucleotide exchange factor (GEF) proteins and specifically targeted Rac1 GAP [16]. As the important roles of SH3BP1 and SH3BP1-Rac1 pathway in human cancer is emerging in recent studies [17, 18]. Moreover, it is not clear whether the mechanism underlying SH3BP1 regulated Rac1-WAVE2 signaling in HCC metastasis remains unknown so far. The involvement of SH3BP1 in Rac1-WAVE2 signaling regulation is a question of interest to HCC aggressiveness research, and we hypothesize that SH3BP1 may be a novel tumor-associated SH3 domain gene in HCC.

In the present project, the expression patterns of SH3BP1 in human HCC tissues and several cell lines were determined. To elucidate the functions of SH3BP1 in HCC metastasis, the regulation of SH3BP1 on HCC cell migration and metastasis process was characterized. In translational studies, the clinical values of SH3BP1 serving as a novel WAVE2 regulator and a prognostic biomarker were evaluated to predict future metastasis and recurrence in HCC patients.

## RESULTS

### Elevated SH3BP1 expression levels was associated with HCC metastases

**Figure 1:**
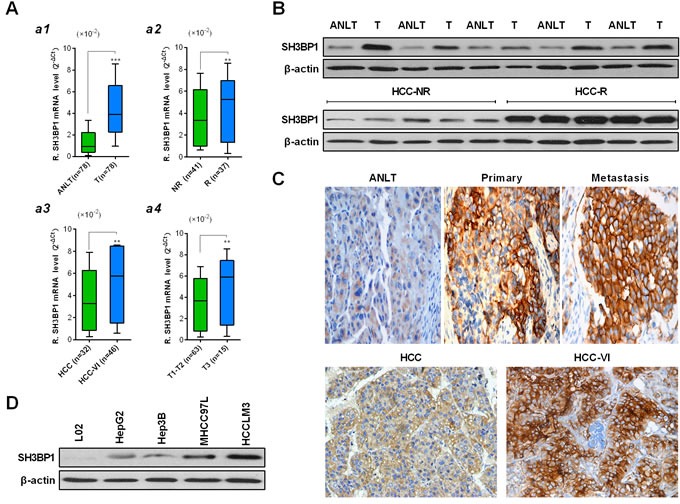
Characteristic of SH3BP1 expression in HCC samples **A.** Real-time RT-PCR analysis was performed in clinical training cohort 1 (fresh-frozen paired HCC tissue samples, *n* = 78), (*a1*) The average expression level of SH3BP1 mRNA in HCC tissues (T) was higher than that in adjacent non-tumorous liver tissues (ANLT), paired Student's t-test; (*a2*) Patients suffering HCC recurrence (R, intrahepatic or extrahepatic metastases) exhibited higher expression of SH3BP1 in tumor tissues than those no recurrences (NR); (*a3*) HCC tissues with vascular invasion (VI) exhibited higher SH3BP1 mRNA expression levels in than those HCC tissues without VI; (*a4*) A significant correlation is demonstrated between increased SH3BP1 mRNA expression and tumor stage. Gene expression results were normalized to internal control β-actin. Data are presented as mean ± SD of 3 independent experiments. *P < 0.05, **P < 0.01, ^***^P < 0.001. **B.** Representative images of Western blots showing relative SH3BP1 protein levels in HCC is higher than ANLT, a significant difference expression of SH3BP1 protein between HCC-R with HCC-NR. **C.** Example of SH3BP1 protein expression in ANLT, primary, metastatic and HCC-VI tissues detected by IHC staining (original × 400 magnification). **D.** Representative images of Western blots showing SH3BP1 in HCC cell lines sample, Relative SH3BP1 protein levels in HCCLM3 is highest than other cell lines. β-actin was used as a loading control.

qRT-PCR analysis indicated that SH3BP1 mRNA was readily detectable in all HCC and paired ANLT tissues of 78 clinical cases. A significant up-regulation of SH3BP1 mRNA expression was identified in HCC compared with ANLT tissues (Figure 1a1, 0.0434 ± 0.0022 *vs.* 0.0095 ± 0.0011; *P* < 0.001). The patient patients without recurrences exhibited slightly SH3BP1 mRNA expression levels than those with HCC recurrence (Figure 1a2, 0.0387 ± 0.0076 *vs.* 0.0599 ± 0.0048, *P* < 0.05). Meanwhile, HCC tissues with vascular invasion (HCC-VI) expressed significantly higher levels of SH3BP1 mRNA than HCC tissues without VI (Figure 1a3, 0.0792±0.0059 *vs.* 0.0368 ± 0.0073, *P* < 0.01). HCC of T3 stage exhibited a significantly higher SH3BP1 mRNA expression than that of T1-T2 stages (Figure 1a4, 0.0676 ± 0.0093 *vs.* 0.0357 ± 0.0057, *P* < 0.01). However, there was no significant association between SH3BP1 mRNA expression and other clinicopathologic parameters, such as age, gender, liver cirrhosis, serum AFP, tumor diameter, tumor encapsulation (data not shown).

The qRT-PCR results were further verified by IHC staining and Western blot of the same set of human specimens and HCC cell lines. As shown in Figure 1B & 1C, the results of SH3BP1 protein detection and comparison between HCC and ANLT tissues, HCC with recurrence and without recurrence tissues, primary and metastatic HCC tissues, HCC and HCC-VI tissues were consistent with that in SH3BP1 mRNA measurements. To validate the characterization of SH3BP1 expression in HCC, the expression of SH3BP1 in four HCC cell lines with varied metastasis potential was confirmed by qRT-PCR analysis (Supplemental Figure S1) and Western blot (Figure 1D). HCCLM3 cells were demonstrated to have the highest SH3BP1 protein expression than the other three HCC cell lines of HepG2, Hep3B, MHCC97L and an immortalized liver cell line of L02.

### SH3BP1 enhanced HCC cell metastasis but not cell growth *via* Rac1 activation *in vitro* and *in vivo*

**Figure 2:**
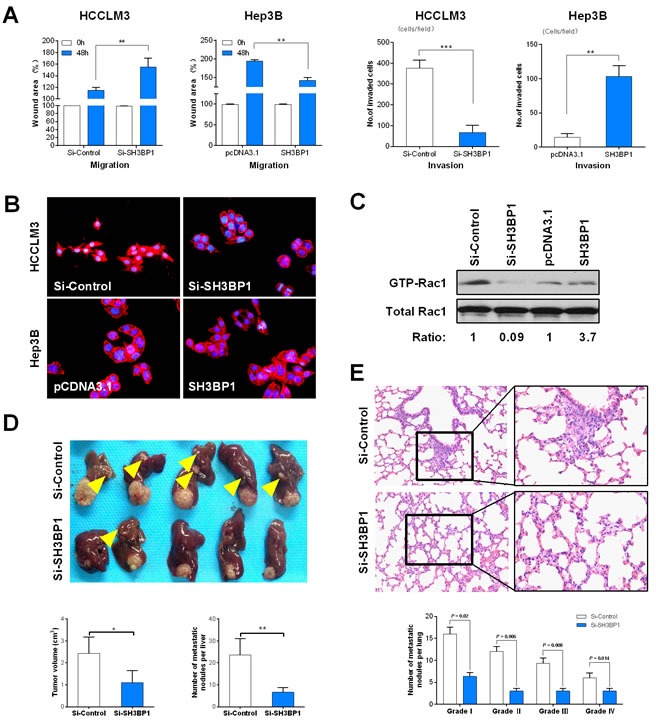
SH3BP1 promotes HCC cell invasion and metastasis *in vivo* and *in vitro* through Rac1-WAVE2 pathway activation **A.** Wound healing analysis showed a significant difference in the speed between the SH3BP1 knockdown (Si-SH3BP1-HCCLM3) and over expression SH3BP1 (SH3BP1-Hep3B) group cells compared with the negative control (left). Boyden chamber invasion analysis showed a significant difference lower number of invasive cells in Si-SH3BP1 HCCLM3 and Hep3B cells than HCCLM3 and SH3BP1 Hep3B cells (right). **B.** Cytoskeletal rearrangement events were decreased in SH3BP1-depleted HCCLM3 cells (top) and were increased in ectopically expressed SH3BP1 Hep3B cells (bottom). **C.** GTPase pull-down and immune-blot assays showing that endogenous SH3BP1 depletion in HCCLM3 cells corresponded to a decrease in the amount of GTP-bound activate Rac1, whereas the level of total Rac1 remained unchanged. Correspondingly, ectopic expression of SH3BP1 in Hep3B cells readily induced an increase in active GTP-Rac1 level without affecting the total Rac1 level. The relative signal of each band in the GTP-bound form of the pull-down experiments was normalized to the total amount detected in the whole-cell lysates and followed by normalization to the untreated control of the same cell lysate. **D.** The morphologic characteristics of tumor in Si-SH3BP1 and Si-Control groups (top). The comparison of tumor size and the number of intra-hepatic metastasis nodules between Si-SH3BP1 and Si-Control groups (bottom). **F.** The characteristics of lung metastases detected by H&E staining (top). Lung metastasis rates between Si-SH3BP1 and Si-Control groups are statistically different in each grade from I to IV (bottom).

To investigate the functional roles of SH3BP1 in HCC, we respectively depleted SH3BP1 expression by siRNA in HCCLM3 cells and transfected Hep3B cells with SH3BP1 *in vitro*. Boyden chamber invasion and wound healing assays demonstrated that SH3BP1 significantly enhanced the migration and invasion abilities of HCC cells (Figure 2A & Supplemental Figure S2, *P* < 0.01). IF staining revealed greatly reduced F-actin polymerization and stress fiber disassembly in SH3BP1-depleted HCCLM3 cells, and increased actin cytoskeleton rearrangements in Hep3B cells ectopically expressed SH3BP1 (Figure 2B). However, SH3BP1 depletion in HCCLM3 cells and SH3BP1 transfection in Hep3B cells did not exert any significant effect on cell viability detected by cell count and colony-forming, nor cell proliferation measured by cell cycle and apoptosis assay (data not shown).

The activation of guanosine triphosphatase (GTPase) was regarded as a critical regulatory event in actin cytoskeleton rearrangements in HCC cells [19]. Figure 2C showed that SH3BP1 depletion obviously impaired GTPase activity Rac1 in Si-SH3BP1 group (fold ratio = 0.09), although the levels of total Rac1 remained constant in HCCLM3 cells. Correspondingly, increased activation of Rac1 induced by SH3BP1 overexpression was observed in Hep3B cells, as compared with that in the control cells (fold ratio = 3.7), but not of RhoA, Cdc42 and RhoC (data not shown). The level of Rac1 and WAVE2 mRNA expression was confirmed by qRT-PCR analysis (Supplemental Figure S3). These data indicated that SH3BP1 might promote cell motility and invasion of HCC cells *via* regulation of Rac1 activity. In nude mice with HCCLM3 tumor transplantation, the lesion size in the Si-SH3BP1 group was significantly smaller than that in controls (Figure 2D: Top panel, 1.12 ± 0.85 *vs.* 2.45 ± 0.97 cm^3^, *P* < 0.05). Meanwhile, numbers of both intrahepatic metastatic nodules (Figure 2D: Bottom panel, 6 ± 2.2 *vs.* 23 ± 9.3, *P* < 0.01) and pulmonary metastatic nodules (Figure 2E, 17 ± 4.5 *vs.* 53.5 ± 5.6, *P* < 0.01) were significantly decreased by SH3BP1 knockdown. These results identify SH3BP1 as a critical modulator of metastasis of HCC cells *in vivo*.

### SH3BP1 facilitated HCC invasion and metastasis *via* Rac1-WAVE2 pathway

**Figure 3:**
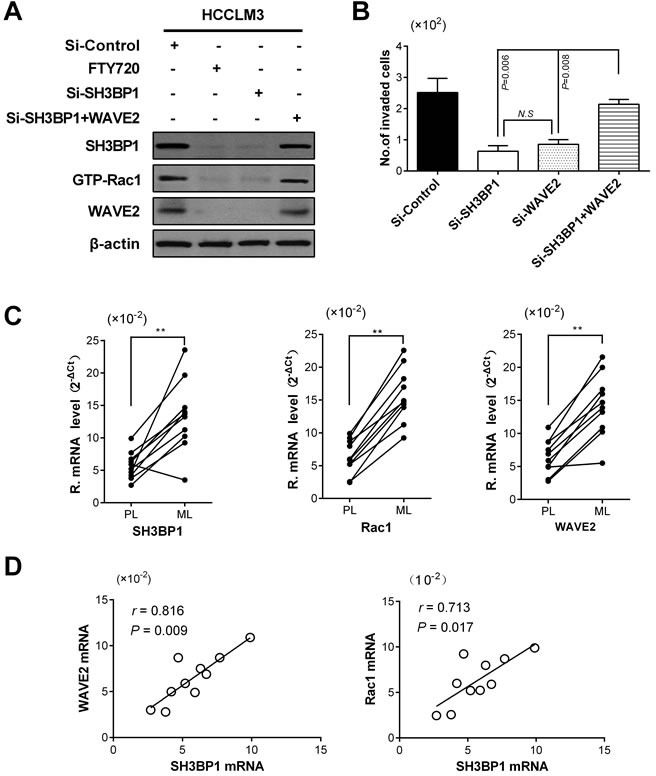
SH3BP1 facilitated HCC invasion and metastasis *via* Rac1-WAVE2 pathway **A.** SH3BP1-depleted and Si-Control HCCLM3 cells whole-cell lysates was measured by Western blotting. No signal for WAVE2 was detected in the whole-cell lysates when SH3BP1 depletion but Rac1 specifically suppressed alone by FTY720. **B.** WAVE2-overexpressed SH3BP1-depleted HCCLM3 transfected with WAVE2 plasmid in Si-SH3BP1 group exhibited behaviors of cell motility and invasion similar to the cells in Si-Control group *in vitro* (*P* < 0.05). **C.** Comparison of SH3BP1, Rac1, and WAVE2 expressions between paired PL and ML tissues (*n* = 10). Relative mRNA levels of these genes were significantly increased in the ML tissues. Statistical analyses were carried out using a paired Student's t-test, **P < 0.01. **D.** A positive correlation was found between SH3BP1 expression, Rac1 expression and WAVE2 expression (*r* = 0.713, *P* = 0.017; *r* = 0.816, *P* = 0.009, respectively). PL: primary lesions; ML: Intrahepatic metastasis lesions.

A specific Rac1 inhibition reagent of FTY720 could suppress HCC cells motility by down-regulating Rac-GTP level [20]. To investigate if Rac1-WAVE2 signaling was involved in SH3BP1-induces motility and invasion of HCC cells, the whole cell lysate of HCCLM3 cells in Rac1 inhibition group was detected by Western blot. Being the same as that, Rac1 expression was specifically suppressed alone by FTY720, no signal of WAVE2 and SH3BP1 was detected in the cell lysate of SH3BP1-depleted HCCLM3 cells (Figure 3A). The level of mRNA expression was confirmed by qRT-PCR analysis (Supplemental Figure S3).

To answer the interrelationships between WAVE2 and SH3BP1 in the regulation of HCC cell invasion, the effects of WAVE2 overexpression in HCCLM3 cells with SH3BP1 knockdown were further examined. Consistently, down-regulated SH3BP1 but overexpressed WAVE2 mimicked the phenotype of strong cell invasion ability of HCCLM3 cells highly expressed SH3BP1 in Si-Control group (Figure 3B). These results supported that Rac1 and WAVE2 were likely to be downstream responder of SH3BP1.

Further qRT-PCR performed in paired primary lesions (PL) and intrahepatic metastasis lesions (ML) demonstrated that ML tissues had higher SH3BP1, Rac1 and WAVE2 expression levels than PL tissues (Figure 3C, *P* < 0.01, respectively). The expressions of SH3BP1 mRNA were significantly correlated with Rac1 and WAVE2 mRNA expression in clinical HCC tissues (Figure 3D). Our results indicated that SH3BP1 acted as a genuine partner and upstream regulator of Rac1 and WAVE2, and the active pathway of SH3BP1-Rac1-WAVE2 were involved in HCC metastasis.

### SH3BP1 mediated VEGF expression in HCC cells

**Figure 4:**
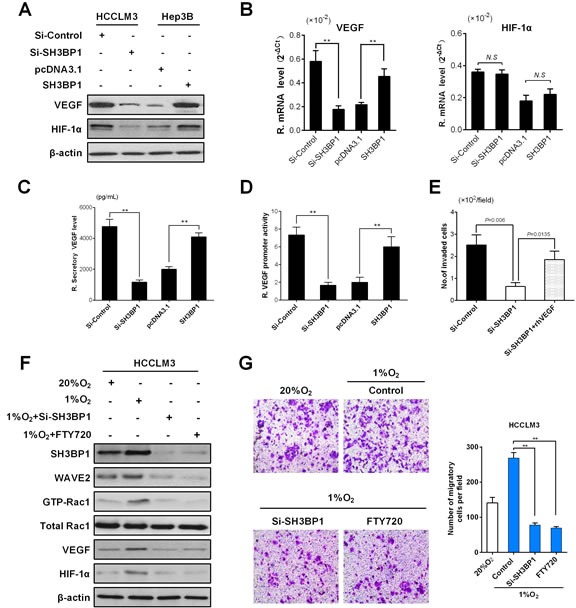
SH3BP1 induces VEGF secretion, activation of VEGF promoter and increase HIF-1α expression enhanced HCC aggressiveness **A.** Using Western blot assay, accompanied by depleted SH3BP1 protein levels, VEGF and HIF-1α protein levels decreased in HCCLM3 cells. On the contrary, the evidence showed that decreased VEGF and HIF-1α protein levels when compared with SH3BP1-Hep3B and parental Hep3B cell lines. **B.** VEGF mRNA levels (left), but not HIF-1α mRNA levels (right), were changed in Si-SH3BP1-HCCLM3 or ectopic SH3BP1 Hep3B cells when compared with controls by qRT-PCR analysis. **C.** At the protein level, the secretion of VEGF was significantly increased in HCCLM3 and ectopic SH3BP1-Hep3B group was measured by ELISA. **D.** Full lengths of VEGF promoter were employed for the examination of the promoter activity on SH3BP1 activation in each group HCC cells. **E.** Si-SH3BP1-HCCLM3 cells could be elevated by the addition of exogenous rhVEGF (20ng/mL) when compared with Si-SH3BP1 and parental HCCLM3 cell lines using Matrigel invasion assay. **F.** HCCLM3 cells were treated with Si-SH3BP1 or FTY720 in normoxia (20%O_2_) or hypoxia (1%O_2_). SH3BP1, Rac1 and WAVE2 protein were decreased in Si-SH3BP1-HCCLM3 or FTY720 cells when compared with controls by Western blot analysis. (G) Under normoxia (20%O_2_) or hypoxia (1%O_2_) conditions, HCCLM3 cells were allowed to invade Matrigel for 24 hours (left). The number of invasive cells was quantified (right). Invasion is expressed relative to the levels observed with normoxic controls.

Rac1 activity was documented to be required for hypoxia-inducible factor 1α (HIF-1α) activation and VEGF expression elevation, which promoted angiogenesis, microscopic venous invasion, and tumor invasiveness of HCC [21]. Being consistent with this report, our data observed down-regulated protein expression of VEGF and HIF-1α in HCCLM3 cells infected with Si-SH3BP1 lentivirus, but up-regulated VEGF and HIF-1α protein expression in Hep3B cells with SH3BP1 transfection (Figure 4A). However, qRT-PCR analysis exhibited that only VEGF mRNA levels (Figure 4B: Left), but not HIF-1α mRNA levels, were regulated by SH3BP1 expression in HCC cells (Figure 4B: Right). Applying Enzyme-linked immunosorbent (ELISA) assay, a decreased VEGF protein secretion was found in Si-SH3BP1 HCCLM3, and an increased VEGF protein secretion was identified in SH3BP1-transfected Hep3B, as respectively compared with their control group (Figure 4C, *P* < 0.01, respectively).

To determine if SH3BP1 activated VEGF at the transcriptional level, Si-SH3BP1 and SH3BP1 respectively together with a luciferase reporter harboring full lengths of VEGF promoter was cotransfected into HCC cells to generate a transient transfection. We found that VEGF promoter activity was decreased significantly by 3.6 fold by SH3BP1 silencing in HCCLM3 cells. Instead, SH3BP1 overexpression significantly increased VEGF promoter activity by 3.2 fold Hep3B cells (Figure 4D, *P* < 0.01, respectively). However, an addition of exogenous rhVEGF (20ng/mL) markedly increased the number of invaded HCCLM3 cells with SH3BP1 knockdown in Matrigel invasion chamber assay (Figure 4E, *P* < 0.05). These results revealed the functional significance of SH3BP1 in angiogenesis and metastasis of HCC cells.

### SH3BP1-induced WAVE2 pathway activation is involved in hypoxia-induced cell invasion

HCC tumors are normally characterized by poor oxygenation [22]. Under hypoxic conditions, tumor cells can change their living condition through invading to surrounding tissue or inducing angiogenesis [23, 24]. SH3BP1, Rac1, WAVE2, VEGF, and HIF-1α protein expression was significantly increased as a result of hypoxia. Knockdown of endogenous SH3BP1 expression by using Si-SH3BP1, we found that depletion of SH3BP1 induces down-regulation Rac1, WAVE2, VEGF, and HIF-1α. To determine whether hypoxia-induced SH3BP1 activation is WAVE2 dependent, After SH3BP1 siRNA and ectopic expression of WAVE2 treatment, and the hypoxia-induced expression of cleaved WAVE2 was also significantly diminished (Figure 4F). Under hypoxia, invasion of HCCLM3 cells through reconstituted three-dimensional Matrigel matrices was increased. To elucidate whether SH3BP1-induced Rac1-WAVE2 pathway activation was responsible for the increase in cell invasion observed in hypoxia, HCCLM3 cells were treated with Si-SH3BP1. Blockade of SH3BP1 inhibited the increase in cell invasion observed during hypoxia. HCCLM3 cells treated with the Rac1 inhibitor, FTY720, also exhibited significantly decreased hypoxia-induced invasion (Figure 4G). Collectively, these results demonstrate that SH3BP1-mediated Rac1-WAVE2 pathway activation is required for hypoxia induced invasion in HCC cells.

### The correlations between SH3BP1 expression and pathological stage in HCC

**Figure 5:**
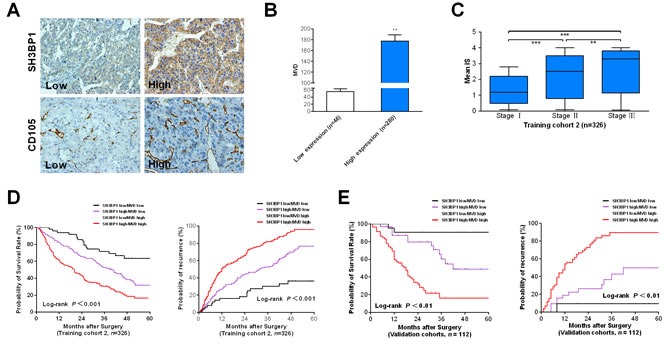
Combination of elevated SH3BP1 expression and MVD count is a powerful simplified predictor model in poor clinical outcome in HCCs **A.** Elevated SH3BP1 expression in training cohort 2 of 326 paired HCC clinical samples were determined by IHC method (Original magnification, ×400). Based on the results of IHC staining, the expression of SH3BP1 was classified as low expression (IHC score 0 & 1+, *n* = 46) and high expression (IHC sores 2+ & 3+, *n* = 280). **B.** MVD in training cohort 2 HCC tissues was determined by CD105 IHC staining as described in Materials and Methods. Students't test shows that SH3BP1 protein high expression group had the higher MVD than low expression group. **C.** According to the scale of IHC staining in training cohort 2, higher IS of SH3BP1 in HCC tumors was significantly associated with tumor advanced tumor stage (*P* < 0.001). **D.** Kaplan-Meier method estimates of overall survival time and time to recurrence (TTR) to training cohort 2 HCC patients. **E.** The predictive value of the simplified model existed in cohort 3 of 112 cases. Low risk group: SH3BP1^Low^/MVD^Low^; Medium risk group: SH3BP1^High^/MVD^Low^ or SH3BP1^Low^/MVD^High^; High risk group: SH3BP1^High^/MVD^High^. Value of *P* < 0.05 was considered statistically significant.

To validate the characteristic association between SH3BP1 expression and HCC, SH3BP1 protein expression was subsequently detected in training cohort II. According to IHC scores of SH3BP1 staining (Figure 5A), 326 HCC cases were divided into low expression group (*n* = 46) and high expression group (*n* = 280). In concordance with the results of the correlation between SH3BP1 expression levels and clinic-pathological indexes obtained in training cohort I, there was a significant association between high SH3BP1 expression and tumor number (*P* = 0.036), the presence of VI and histological TNM stages in training cohort II (Table 1, *P* < 0.001). Meanwhile, MVD count determined by CD105 staining in HCC tissues indicated that high SH3BP1 expression group had a significantly higher MVD than that in low SH3BP1 expression group (Figure 5B, 178.6 ± 26.2 *vs.* 56.3 ± 12.7, *P* < 0.01, respectively).

**Table 1:** Correlations between SH3BP1 expression and clinical and pathological indexes in training cohort 2 of 326 HCC cases

Clinical and pathological indexes		SH3BP1 protein level			
		Low (%)	High (%)	R (high / low)	*P*
Age, years	≤60	25 (54.3)	168 (60.0)	6.7	0.470
	>60	21 (45.7)	112 (40.0)	5.3	
Sex	Male	39 (84.8)	255 (91.1)	6.5	0.184
	Female	7 (15.2)	25 (8.9)	3.5	
HBsAg	Negative	6 (13.0)	47 (16.8)	7.8	0.524
	positive	40 (87.0)	233 (83.2)	5.8	
Albumin	≤35 g/L	43 (93.5)	252 (90.0)	5.7	0.456
	>35 g/L	3 (6.5)	28 (10.0)	9.3	
Child-Pugh classification	A	45 (97.8)	277 (98.9)	6.2	0.529
	B	1 (0.22)	3 (1.1)	3.0	
Preoperative AFP (ng/mL)	≤20	24 (52.2)	143 (51.1)	6.0	0.890
	>20	22 (47.8)	137 (48.9)	6.2	
Liver cirrhosis	Absent	3 (6.5)	32 (11.4)	10.7	0.319
	Present	43 (93.5)	248 (88.6)	5.8	
Tumor encapsulation	Complete	21 (45.7)	117 (41.8)	5.6	0.623
	None	25 (54.3)	163 (58.2)	6.5	
Tumor size (cm)	≤5	20 (43.5)	99 (35.4)	5.0	0.289
	>5	26 (56.5)	181 (64.6)	7.0	
Tumor number	Single	19 (41.3)	162 (57.9)	8.5	0.036
	Multiple*	27 (58.7)	118 (42.1)	4.4	
Vascular invasion	Absent	33 (71.7)	123 (43.9)	3.7	<0.001
	Present ^§^	13 (28.3)	157 (56.1)	12.1	
MVD	≤65.5	32 (69.5)	125 (44.6)	3.2	<0.001
	>65.5^#^	14 (30.5)	155 (55.4)	11.9	
Edmondson-Steiner grade	I-II	35 (76.1)	218 (77.9)	6.2	0.790
	III-IV	11 (23.9)	62 (22.1)	5.6	
TNM stage	I	34 (73.9)	119 (58.6)	3.5	<0.001
	II-III	12 (26.1)	161 (41.4)	13.4	

Abbreviations: HCC, hepatocellular carcinoma; HBsAg, hepatitis B surface antigen; AFP, a-fetoprotein; TNM, tumor-node-metastasis staging

* Multiple was defined as: tumor number > 2.

§ Vascular invasion (VI) was defined as: gross invasion and microscopic invasion involving portal vein, hepatic vein, inferior vena cava (IVC).

# Considering the data of MVD count do not fit the normal distribution, the median value of 65.5 (but not the mean value) is used as the cut-off for low and high MVD.

Moreover, a significant correlation was verified between SH3BP1 expression and MVD (*R* = 0.823, *P* < 0.01), revealing the significance of increased SH3BP1 expression in HCC tumor angiogenesis. To further investigate the clinical impact of up-regulated SH3BP1 in angiogenesis of HCC, we subsequently divided 326 HCC cases into HCC group (HCC tissue without VI, *n* = 156) and HCC-VI group (HCC tissue with VI, *n* = 170) based on the clinical and pathological indexes. High SH3BP1 expression in HCC tumors was found to be significantly associated with tumor VI and pathological HCC stage (Figure 5C).

### Relationship between SH3BP1 expression and HCC poor prognosis

In multivariable Cox regression analysis in training cohort 2 (Table 2), high serum AFP level (risk ratio [RR], 1.76; *P* = 0.041), high SH3BP1 expression (RR, 1.69; *P* = 0.008), MVD (RR, 1.43; *P* = 0.003), TNM stage (RR, 1.59; *P* = 0.045), and VI (RR, 1.32; *P* = 0.017) were found to be independent prognostic factors for overall survival (OS) and time to recurrence (TTR). The predict model based on SH3BP1 expression combined with MVD was an independent predictor of OS and TTR in training cohort 2. A predictive risk score model based on SH3BP1 expression levels in combination with MVD count was constructed (Figure 5D). All HCC subjects in training cohort II were divided into three groups: I (Low risk group, *n* = 59), SH3BP1^Low^/MVD^Low^; II (Medium risk group, *n* = 150), SH3BP1^High^/MVD^Low^ or SH3BP1^Low^/MVD^High^; III (High risk group, *n* = 117), SH3BP1^High^/MVD^High^. As shown in Figure 5D. The differences of 1-, 3-, 5-year OS and TTR between each two groups were statistically significant (*P* < 0.05, respectively).

**Table 2:** Multivariable Cox regression analysis of overall survival in training cohort 2 of 326 HCC cases

Clinical and pathological indexes	*n*	Univariable analysis		Multivariable analysis	
		RR (95% CI)	*P*	RR (95% CI)	*P*
Albumin (g/L)					
>35	295	1		1	
≤35	31	1.73 (1.19-2.32)	0.027	1.73(1.02-2.53)	0.052
Child-Pugh classification					
A	322	1		1	
B	4	1.69 (1.07-2.51)	0.018	1.58(1.04-2.49)	0.066
Preoperative AFP level (ng/mL)					
≤ 20	167	1		1	
> 20	159	1.82 (1.12-2.58)	0.035	1.76(1.02-2.53)	0.041
Tumor nodule number					
Single	181	1		1	
Multiple *	145	1.62 (1.07-2.76)	0.028	1.56(1.05-2.57)	0.063
Tumor encapsulation					
Presence	138	1		—	—
Absence	188	1.72 (1.04-2.83)	0.033	—	—
Edmondson-Steiner grade					
I – II	253	1		—	—
III – IV	73	1.72 (1.03-2.87)	0.037	—	—
Vascular invasion^§^					
Absence	156	1		1	
Presence	170	1.75 (1.15-2.65)	0.009	1.32(1.01-2.38)	0.017
TNM stage					
I	153	1		1	
II–III	173	1.65 (1.09-2.52)	0.019	1.59(1.01-2.50)	0.045
SH3BP1 expression					
Low	46	1		1	
High	280	1.97 (1.28-3.09)	0.003	1.69(1.02-2.72)	0.008
MVD					
≤65.5	137	1		1	
> 65.5	189	1.62 (1.04-2.53)	0.028	1.43(1.02-2.33)	0.003

Abbreviations: RR, risk ratio; HBsAg, hepatitis B surface antigen; AFP, a-fetoprotein; TNM, tumor-node-metastasis staging; MVD, microvessel density.

* Multiple was defined as: tumor number > 2.

§ Vascular invasion (VI) was defined as: gross invasion and microscopic invasion involving portal vein, hepatic vein, inferior vena cava (IVC).

### The prognosis prediction value of SH3BP1 expression in HCC patients

To explore if SH3BP1 combined with MVD was an independent clinical predictor of HCC outcome, the predictive value of this combined parameter was evaluated in another independent validation cohort of 112 HCC patients. In the validation model, 21/112 patients were categorized as high risk, 54/112 patients as medium risk, 37/112 patients and as low risk. In accordance with that found in training cohort II, there were significant differences of OS and TTR between each two groups (*P* < 0.05, Figure 5E). This validation suggested an important role of SH3BP1 relevant to HCC recurrence and confirmed the clinical value of SH3BP1 expression status in HCC prognosis prediction.

## DISCUSSION

SH3BP1 inhibits Rac1 activity in cancer cell motility [21, 25]. The present study firstly determined significantly increased SH3BP1 expression in most primary HCC, HCC-VI, metastatic and recurrent HCC tissues. Among several HCC cell lines with different metastasis potentials, the highest SH3BP1 expression found in the most metastatic HCC cells of HCCLM3 suggested an association between SH3BP1 overexpression and metastasis potential of HCC. Conversely, depletion of endogenous SH3BP1 in high metastasis potential cells significantly reduced the mobility and invasiveness of HCCLM3 cells. In concordance with the knockdown studies, ectopic expression of SH3BP1 in Hep3B promoted cell migratory and invasive abilities, indicating that SH3BP1 is closely involved in HCC invasion.

SH3BP1 protein overexpression in human HCC was found to be significantly correlated with VI, which is a central clinicopathological feature of metastasis and poor prognosis of HCC. The data obtained from GTP pull-down assay, immunoblotting assay and dual-luciferase reporter system assay demonstrated that SH3BP1 induced an activation of Rac1, and elevated WAVE2, VEGF and HIF-1α activity. In the immunoblotting of both SH3BP1-depleted and Rac1-suppressed HCCLM3 cells by FTY720, suggested SH3BP1 as a genuine Rac1 and WAVE2 partner. Moreover, SH3BP1 promoted HCC angiogenesis not only through elevation of VEGF level as well as secretion. SH3BP1 promotes angiogenesis and then contributes on cancer development and metastasis.

MVD and vessel maturation are two angiogenesis markers in surgical pathology [26]. The presence of VI in pathological specimens after liver resection is a well-known unfavorable prognostic factor for HCC recurrence [27]. The association between SH3BP1 expression and the clinicopathologic characteristics in HCC subjects revealed that SH3BP1 expression was significantly correlated with VI of HCC. HCC patients with high SH3BP1 expression indicate worse prognosis. Moreover, a multivariable Cox regression analysis indicated that both SH3BP1 and MVD were independent prognostic indicators of HCC OS and TTR. A predictive risk score model based on SH3BP1 expression combined with MVD count was constructed and provided us more refined and systematic stratification for future risk prediction of HCC recurrence.

**Figure 6:**
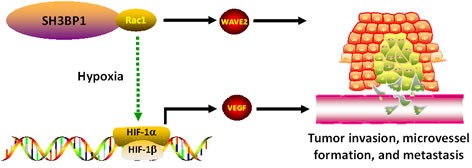
A illustration for SH3BP1 in Rac1 activity modulation

Rac1 is most important regulator in cancer invasion and metastasis, while the efficient inhibitor is not applied in clinical even extensively investigation were done. That suggested the potential role of Rac1 is largely remaining unclear. Our finding recovered SH3BP1, which binds to Rac1 and modulates its activity and regulates its downstream genes, such as WAVE2 (Figure 6). This study could be valuable to clarify the Rac1 role in cancer.

In conclusion, SH3BP1 induces Rac1-WAVE2 signaling to promote tumor vascular invasion and MVD formation related to HCC metastasis. SH3BP1 overexpressed in tumor is significantly correlated to poor prognosis of HCC. SH3BP1 is a novel Rac1-WAVE2 pathway regulator, a prognostic marker and a potential therapeutic target of HCC.

## MATERIALS AND METHODS

### Patient samples and tissue specimens of HCC

The patient signed a written informed consent form, approved by the local Ethical Committee (Xiangya Hospital or Provincial Tumor Hospital, Central South University, China). HCC was diagnosed according to World Health Organization criteria. All of clinical specimens were confirmed as HCC by histological diagnosis. None of the patients had received any kind of anticancer treatment prior to surgery, such as transarterial chemoembolization (TACE) or radiofrequency ablation (RFA).

The study comprised three independent cohorts of HCC patients recruited from two medical institutions:i) training cohort 1 (fresh-frozen tissues for qRT-PCR and Western blot analysis, n=78). Matched fresh specimens of HCC and adjacent nontumoral liver tissues (ANLT) were randomly collected from HCC patients receiving hepatic resection in the Department of Hepatobiliary Surgery between July 2010 and March 2011 at Xiangya Hospital, China. The paired fresh tissues were snap frozen in liquid nitrogen and stored at −80°C until use.ii) training cohort 2 (paraffin-embedded livers tissues for IHC analysis, n = 326). The training cohorts were randomly collected from HCC patients undergoing curative hepatic resection from September 2001 to June 2004 in Xiangya Hospital. The paired tumor and ANLT tissues were partly embedded in paraffin after fixation in 10% formalin for histological diagnosis and IHC analysis. iii) validation cohort 3 (paraffin-embedded livers tissues for IHC analysis, n = 112). The validation cohort comprised of 112 randomly selected patients with HCC who underwent curative hepatic resection between October 2005 and March 2006 at Hunan Provincial Tumor Hospital.

The detailed inclusion clinicopathological criteria were defined as previously described. We also recorded platelet count, serum albumin level, total bilirubin level, Child-Pugh score, model for end-stage liver disease (MELD) score, alpha-fetoprotein (AFP) level, number of nodes, tumor size, satellite nodules, Barcelona clinic liver cancer (BCLC) stage and pathological tumor-node-metastasis (TNM) classification (6th Edition). Data with which to assess the prognostic impact of lymph node metastasis were insufficient and thus N status was not evaluated in the present study.

### Collection of follow-up data

The Follow-up was terminated on 26 June 2011. Postsurgical patient surveillance was performed through telephone, home visits, and return visits to the outpatient clinic to achieve a follow-up rate of 97.1% (438/451). Follow-up time ranged from 2 to 63 months, with a median follow-up time of 38.5 months. Deaths from other causes were treated as censored cases. For surviving patients, the data were censored at the last follow-up. Once the evidence of recurrence was confirmed by clinical examination, serial AFP level mensuration, ultrasonography or computed tomography (CT) scan was performed. Endpoints were defined as overall survival (OS) and time to recurrence (TTR). The Cox proportional hazards model was used to find hazard ratios (HR) and 95% confidence intervals (CIs) for these clinical endpoints. All procedures in this prognostic study strictly complied with the REMARK guidelines for reporting prognostic biomarkers in cancer.

### Cell lines and culture conditions

MHCC97L and HCCLM3 cell lines were purchased from Liver Cancer Institute of Fudan University, Shanghai, China. The cell lines of L02, HepG2, Hep3B and HMVEC were purchased from American Type Culture Collection (ATCC, Manassas, VA). The cells were cultured in low glucose Dulbecco's Modified Eagle Media (DMEM, GIBCO, Gaithersburg, MD) supplemented with 10% fetal bovine serum at 37°C under an atmosphere of 95% air and 5% CO_2_. For hypoxic exposure, cells were placed in a hypoxia chamber in an atmosphere consisting of 94.9% N_2_, 5%CO_2_, and 0.1% O_2_.

### Total RNA extraction and quantitative real-time PCR (qRT-PCR)

Total RNA extraction, complementary DNA (cDNA) synthesis, and qRT-PCR with SYBR Green fluorescent-based assay were performed per the kits manufacturer's instructions (Takara Bio, Otsu, Japan) on an ABI7300 real-time thermal cycler (Applied Biosystems, Foster City, CA). The detailed specific oligonucleotide sequences of qRT-PCR primers are given in Supplementary Table S2. Glyceraldehyde-3-phosphate dehydrogenises (GAPDH) or β-actin was used as an internal control. The relative messenger RNA (mRNA) expression levels in tissue samples and cell lines were calculated by 2^−ΔCt^ method based on the threshold cycle (Ct) values and were normalized to the internal control GAPDH or β-actin and calculated by 2^−ΔCt^ method.

Preparation of protein lysate and Western blotting

Total protein was extracted from tissues and cells lines on ice using RIPA lysis buffer (Sigma #R0278, St Louis, MO) containing protease and phosphatase inhibitor cocktails (Sigma #P2850, P8340, P5726). Protein concentration was quantified using a Bradford assay kit (Thermo Fisher Scientific Pierce, Rockford, IL). For immunoprecipitation, a total of 100 μg protein was incubated with l μg of anti-SH3BP1 antibodies (Thermo #PA5-18249). The membranes were respectively incubated with the primary antibodies at 4°C overnight followed by HRP-conjugated secondary antibodies (KPL, Gaithersburg, MD. 1:3000 dilution) for 1 hour at 37°C. The antibody-antigen complexes were visualized using Chemiluminescence Luminol Reagent (Thermo Fisher Scientific Pierce). The signals on immunoblotting gels were quantified using BandScan software (Bio-Rad Laboratories, Hercules, CA) and defined as the ratio of target protein to β-actin (Sigma). The detailed information of the primary antibodies and working dilution are given in Supplementary Table S3.

### IHC staining and MVD scoring

Following deparaffinization, antigen retrieval was achieved by microwave treatment with EDTA buffer (1mM, pH 8.0). 4 μm-thick formalin-fixed paraffin tissue sections were incubated at 37°C with the primary antibody overnight at 4°C. The slides were next incubated with the anti-rabbit or anti-mouse secondary antibody conjugated with streptavidin-biotin-peroxidase complex (LSAB2/HRP kit; DAKO A/S, Glostrup, Denmark), and a color reaction was developed using 3, 3-diaminobenzidine tetrahydrochloride (Sigma). The details of the primary antibodies and work dilutions are given in Supplementary Table S3. The negative control slides were probed using normal serum with the same origin as the primary antibodies under the standard experimental conditions. The intensity of SH3BP1 protein staining in HCC specimens was classified using a four-point scale according to the percentage of positive-staining hepatocytes (0: ≤10%, 1+: 11-25%, 2+: 26-50%, 3+: ≥51%), so that the expression levels of SH3BP1 protein were thus divided into low expression (0 or 1+) and high expression categories (2+ or 3+).

To assess tumor microvessels, the sections were stained with CD105 monoclonal antibody for IHC measurement. To count the average microvessels number, any brown-stained endothelial cell or endothelial cell cluster that was clearly separated from adjacent microvessels, tumor cells, and connective elements was counted as 1 microvessel, irrespective of the presence of a vessel lumen. In each case, the immunoreactivity was determined in five random fields and the count was made independently by two experienced pathologists (Dr. Liang Zeng & Dr. Hongwu Zeng) in a blind fashion as previously described.

### Infection of recombinant lentivirus

In SH3BP1 knockdown experiments, pLKO.1-SH3BP1 short-hairpin RNA (shRNA) lentiviral vector was constructed. Three pairs of putative candidate sequences, each had unique 21bp oligonucleotides derived from mRNA transcript of human SH3BP1 gene (NM_018957.3), were designed using Oligoengine software and their specificities were confirmed using nucleotide BLAST searches. One pair of insert sequences containing 21bp oligonucleotides in random sequence without targeting any known gene was used as the control vector for lentivirus infection (Supplementary Table S4). The titer of Si-SH3BP1 lentivirus was 1×10^9^ TU/mL and the titer of Si-Control lentivus was 2×10^9^ TU/mL. The lentivirus was transfected into HCCLM3 cells with an optimal multiplicity of infection (MOI) of 20 TU/cell, which could increase the number of infected cells as well as increase the number of lentivirus copies in each cell. Down-regulation of SH3BP1 expression (at least 80%) was confirmed using qRT-PCR and Western blot.

### Invasion and migration assays

Wound healing assay and Matrigel invasion chamber assay were performed in triplicate as described previously.

### Immunofluorescence (IF) staining of filamentous actin

To visualize the effect of distributions of filamentous actin proteins, Si-Control and Si-SH3BP1 HCCLM3 was fixed with 4% formaldehyde dissolved in PBS for 10 minutes at room temperature and permeabilized for 15 minutes with 0.1% Triton X-100. Cells were incubated in 1% bovine serum albumin in PBS for 30 minutes to block nonspecific antibody binding, and then incubated with 1 μg/mL FITC phalloidin (Sigma Chemical Co., St. Louis, MO) overnight at 37°C. The slides were analyzed by an image analysis system.

### Detection of Rac1 activation

HCCLM3 and Hep3B cells transfected with Si-SH3BP1 or SH3BP1 were serum-starved overnight prior to experiment. The activation of Rac in HCC cells was detected as previously described. The fold ratio obtained was then compared with the fold ratio of the corresponding control group cell sample which was normalized to 1. These experiments were performed in triplicate.

### Dual-luciferase promoter reporter assay

Full-length VEGF promoter cDNA was subcloned into pGL3 by standard protocol. The following plasmids were used: pGL3-VEGF (Promega, Madison, WI), pRLTK (an internal control plasmid containing the herpes simplex thymidine kinase promoter linked to a constitutively active *Renilla* luciferase reporter gene) and pGL3 (plasmid vector alone as a negative control). HCCLM3 and Hep3B (5 ×10^4^ cells per well) was plated into 24-well culture plates and allowed to grow for 24 hours, and pRL-CMV-Luc were cotransfected with either pcDNA3.1-SH3BP1 or pcDNA3.1 into the cells using Fugene 6 reagent (Roche Diagnostics, Indianapolis, IN) according to the manufacturer's instructions. After cells were incubated in the transfection media for 24 h, the media were changed to serum-free media. The cells were lysed 48 hours after transfection and were assayed for luciferase activity using the Dual-Luciferase Reporter Assay System (Promega, Madison, WI), and luciferase activity was determined with a single sample luminometer, as outlined in the manufacturer's protocol. Firefly luciferase activity was measured 48 hours after transfection and the reading was then normalized with the Renilla luciferase activity, which served as an internal control for transfection efficiency.

### Enzyme-linked immunosorbent (ELISA) assay

The levels of VEGF in collected cell medium were determined using a quantitative sandwich ELISA assay kit (R&D Systems, Inc., Minneapolis, MN) according to the manufacturer's instructions. Briefly, 50 μL of each of the cell culture supernatants were loaded in duplicate onto the microtiter plate and analyzed following the manufacturer's instructions. Final color was quantitated at 450 nm on a FLx800 Fluorescence Microplate Reader (BioTek, Instruments, Inc), VEGF concentration was calculated by interpolation with a standard curve generated with recombinant enzyme, which is included in the kit. Data were obtained from 3 independent experiments.

### Colony-forming assay

HCCLM3 cells were seeded into six-well culture plates at a density of 500 cells/well in triplicate wells. After incubation for 14 days, colonies were stained with cell solution (Millipore) and the numbers of colonies containing more than 50 cells were counted. Average colony density was calculated and expressed as the relative percentage of colonies.

### Cell cycle assay

The cell cycle was analyzed by flow cytometry as described previously. Briefly, 1 × 10^6^ cells were harvested and washed in PBS then fixed in 75% alcohol for 30 minutes at 4°C. After washing in cold PBS 3 times, cells were resuspended in 1 mL PBS solution with 40 μg of propidium iodide (PI) (Sigma-Aldrich Corp), 0.1% Triton X-100, and 100 μg of RNase A (Sigma-Aldrich Corp) for 30 minutes at 37°C. Samples were then analyzed with a FACSCalibur (BD Biosciences). Fragmented apoptotic nuclei were recognizable by their subdiploid (sub-G1) DNA content. The percentage of sub-G1 cells was recorded for each sample.

### Apoptosis assay

The cell apoptosis was also detected by Annexin V-FITC Apoptosis Detection kit according to the protocol outlined by the manufacturer (Beyotime Corp, China). Briefly, after SH3BP1 siRNA, cells were harvested and suspended in a binding buffer (1×). An aliquot of 100 μL was incubated with 5 μL of Annexin V-FITC and 5 μL of PI for 15 minutes in dark and 400 μL binding buffer (1×) was added to each sample. The stained cells were analyzed directly by flow cytometry.

### Statistical analysis

Paired Student-*t* tests were used to compare SH3BP1 mRNA expression levels in fresh HCC tissues. The clinicopathological features in HCC patients with high or low SH3BP1 mRNA expression were compared using Fisher's exact test for categorical variables and Mann-Whitney U test for continuous data presented as mean ± SD. χ^2^ test for proportion and Spearman's correlation assay were used to analyze the relationship between SH3BP1 expression and the clinicopathological characteristics of HCC patients. In cellular experiments *in vitro*, the difference between each two experimental groups was compared using two-tailed Welch's *t* test, unless otherwise indicated. Cox proportional hazards model was employed to determine the relationship between SH3BP1 expression and other clinical variables related to HCC development. The patients in training cohort 2 were divided into three groups (High risk: SH3BP1^High^/MVD^High^, Medium risk: SH3BP1^High^/MVD^Low^ or SH3BP1^Low^/MVD^High^, Low risk: SH3BP1^Low^/MVD^Low^) by SH3BP1 expression level and MVD count. Kaplan-Meier analysis using the Log-rank test was applied to compare the cumulative overall and disease-free survival risks of HCC development in these three groups. To confirm the value to predict HCC recurrence risk, the above model consisting of SH3BP1 expression and MVD count was further evaluated in validation cohort 3. All statistical analyses were conducted with the SPSS for Windows version 13.0 release (SPSS, Inc., Chicago, IL). The value of *P* < 0.05 was considered to be statistically significant.

## SUPPLEMENTARY MATERIAL FIGURES AND TABLES



## References

[1] Siegel RL, Miller KD, Jemal A (2015). Cancer statistics, 2015. CA.

[2] Cervello M, McCubrey JA, Cusimano A, Lampiasi N, Azzolina A, Montalto G (2012). Targeted therapy for hepatocellular carcinoma: novel agents on the horizon. Oncotarget.

[3] Rahbari NN, Mehrabi A, Mollberg NM, Müller SA, Koch M, Büchler MW, Weitz J (2011). Hepatocellular carcinoma: current management and perspectives for the future. Annals of surgery.

[4] Villanueva A, Hoshida Y, Battiston C, Tovar V, Sia D, Alsinet C, Cornella H, Liberzon A, Kobayashi M, Kumada H (2011). Combining clinical, pathology, and gene expression data to predict recurrence of hepatocellular carcinoma. Gastroenterology.

[5] Sugioka K, Sawa H (2012). Formation and functions of asymmetric microtubule organization in polarized cells. Current Opinion in Cell Biology.

[6] Chaffer CL, Weinberg RA (2011). A perspective on cancer cell metastasis. Science.

[7] Nürnberg A, Kitzing T, Grosse R (2011). Nucleating actin for invasion. Nature Reviews Cancer.

[8] Wang SM, Ooi LLP, Hui KM (2011). Upregulation of Rac GTPase-activating protein 1 is significantly associated with the early recurrence of human hepatocellular carcinoma. Clinical Cancer Research.

[9] Yang W-H, Lan H-Y, Huang C-H, Tai S-K, Tzeng C-H, Kao S-Y, Wu K-J, Hung M-C, Yang M-H (2012). RAC1 activation mediates Twist1-induced cancer cell migration. Nature cell biology.

[10] Santibáñez JF, Kocić J, Fabra A, Cano A, Quintanilla M (2010). Rac1 modulates TGF-β1-mediated epithelial cell plasticity and MMP9 production in transformed keratinocytes. FEBS letters.

[11] Yang L-Y, Tao Y-M, Ou D-P, Wang W, Chang Z-G, Wu F (2006). Increased expression of Wiskott-Aldrich syndrome protein family verprolin-homologous protein 2 correlated with poor prognosis of hepatocellular carcinoma. Clinical Cancer Research.

[12] Kurisu S, Suetsugu S, Yamazaki D, Yamaguchi H, Takenawa T (2004). Rac-WAVE2 signaling is involved in the invasive and metastatic phenotypes of murine melanoma cells. Oncogene.

[13] de Kreuk B-J, Hordijk PL (2012). Control of Rho GTPase function by BAR-domains. Small GTPases.

[14] Kessenbrock K, Plaks V, Werb Z (2010). Matrix metalloproteinases: regulators of the tumor microenvironment. Cell.

[15] Sanz-Moreno V, Gaggioli C, Yeo M, Albrengues J, Wallberg F, Viros A, Hooper S, Mitter R, Féral CC, Cook M (2011). ROCK and JAK1 signaling cooperate to control actomyosin contractility in tumor cells and stroma. Cancer cell.

[16] Parrini MC, Sadou-Dubourgnoux A, Aoki K, Kunida K, Biondini M, Hatzoglou A, Poullet P, Formstecher E, Yeaman C, Matsuda M (2011). SH3BP1, an exocyst-associated RhoGAP, inactivates Rac1 at the front to drive cell motility. Molecular cell.

[17] Annibaldi A, Dousse A, Martin S, Tazi J, Widmann C (2011). Revisiting G3BP1 as a RasGAP binding protein: sensitization of tumor cells to chemotherapy by the RasGAP 317-326 sequence does not involve G3BP1. PloS one.

[18] Zhao J, Wu G, Bu F, Lu B, Liang A, Cao L, Tong X, Lu X, Wu M, Guo Y (2010). Epigenetic silence of ankyrin-repeat-containing, SH3-domain-containing, and proline-rich-region-containing protein 1 (ASPP1) and ASPP2 genes promotes tumor growth in hepatitis B virus-positive hepatocellular carcinoma. Hepatology.

[19] Bisi S, Disanza A, Malinverno C, Frittoli E, Palamidessi A, Scita G (2013). Membrane and actin dynamics interplay at lamellipodia leading edge. Current Opinion in Cell Biology.

[20] Lee TK, Man K, Ho JW, Wang XH, Poon RT, Xu Y, Ng KT, Chu AC, Sun CK, Ng IO (2005). FTY720: a promising agent for treatment of metastatic hepatocellular carcinoma. Clinical Cancer Research.

[21] Lee TK, Poon RT, Yuen AP, Man K, Yang ZF, Guan XY, Fan ST (2006). Rac activation is associated with hepatocellular carcinoma metastasis by up-regulation of vascular endothelial growth factor expression. Clinical Cancer Research.

[22] Wilson WR, Hay MP (2011). Targeting hypoxia in cancer therapy. Nature Reviews Cancer.

[23] Zhu AX, Duda DG, Sahani DV, Jain RK (2011). HCC and angiogenesis: possible targets and future directions. Nature Reviews Clinical Oncology.

[24] Quail DF, Joyce JA (2013). Microenvironmental regulation of tumor progression and metastasis. Nature medicine.

[25] Budhu A, Forgues M, Ye Q-H, Jia H-L, He P, Zanetti KA, Kammula US, Chen Y, Qin L-X, Tang Z-Y (2006). Prediction of venous metastases, recurrence, and prognosis in hepatocellular carcinoma based on a unique immune response signature of the liver microenvironment. Cancer cell.

[26] Friedl P, Locker J, Sahai E, Segall JE (2012). Classifying collective cancer cell invasion. Nature cell biology.

[27] Huang TY, Michael S, Xu T, Sarkeshik A, Moresco JJ, Yates JR, Masliah E, Bokoch GM, DerMardirossian C (2013). A novel Rac1 GAP splice variant relays poly-Ub accumulation signals to mediate Rac1 inactivation. Molecular biology of the cell.

