# Supplementary material for: SH3-domain binding protein 1 in the tumor microenvironment promotes hepatocellular carcinoma metastasis through WAVE2 pathway
Source: Oncotarget. 2016 Feb 28;7(14):18356–70. doi: 10.18632/oncotarget.7786 (PMC4951293; doi:10.18632/oncotarget.7786)
Supplement: Supplementary file 1 [file oncotarget-07-18356-s001.pdf]

SH3-domain binding protein 1 in the tumor microenvironment promotes hepatocellular carcinoma metastasis through WAVE2 pathway

Supplementary Material

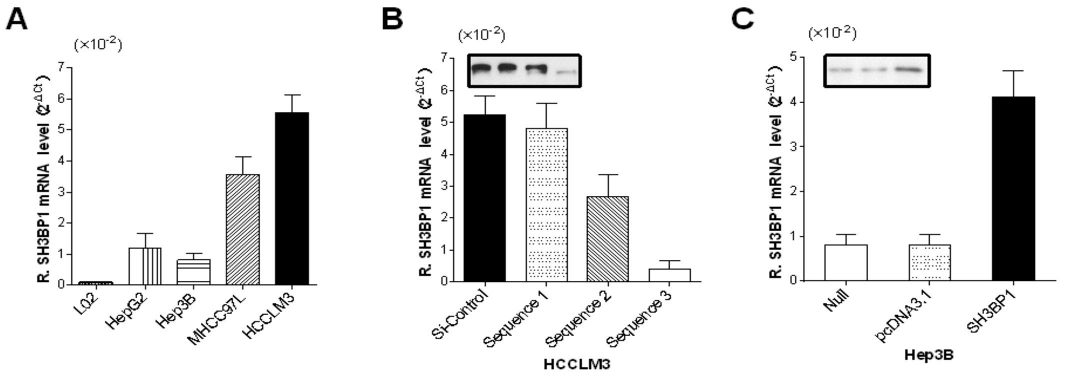

Figure S1: Elevated expression of SH3BP1 mRNA correlates with increased metastatic potential in HCC cells. (A) The expression of SH3BP1 mRNA in four HCC cell lines with varied metastasis potential was confirmed by qRT-PCR analysis. HCCLM3 cells were demonstrated to have the highest SH3BP1 mRNA expression than the other three HCC cell lines of HepG2, Hep3B, MHCC97L and an immortalized liver cell line of L02. (B) Expression of SH3BP1 in infected HCCLM3 cells was measured to evaluate the inhibition efficiency of the three candidate sequences and the control sequence by qRT-PCR and Western blot. (C) Hep3B cells were infected with pcDNA3.1-SH3BP1 to up regulate mRNA and protein expression by qRT-PCR and Western blot.

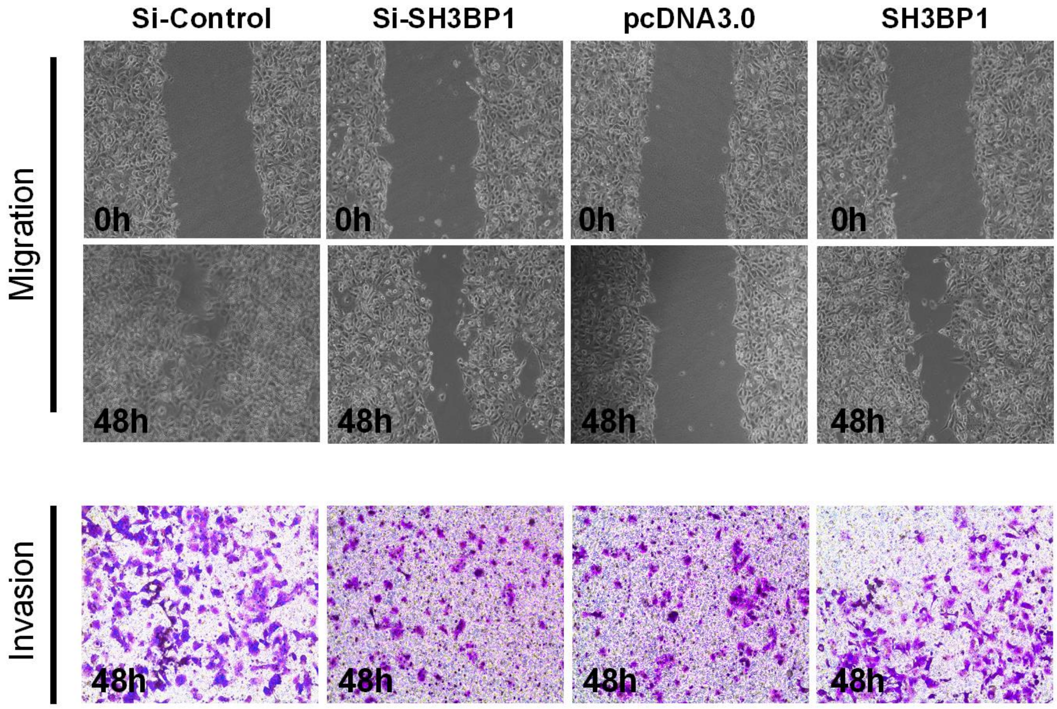

Figure S2: Top panel: Wound healing analysis showed a significant difference in the speed between the SH3BP1 knockdown (Si-SH3BP1-HCCLM3) and over expression SH3BP1 (SH3BP1-Hep3B) group cells compared with the negative control (magnification, ×100). Bottom panel: Boyden chamber invasion analysis showed a significant difference lower number of invasive cells in Si-SH3BP1 HCCLM3 and Hep3B cells than HCCLM3 and SH3BP1 Hep3B cells (magnification, ×200).

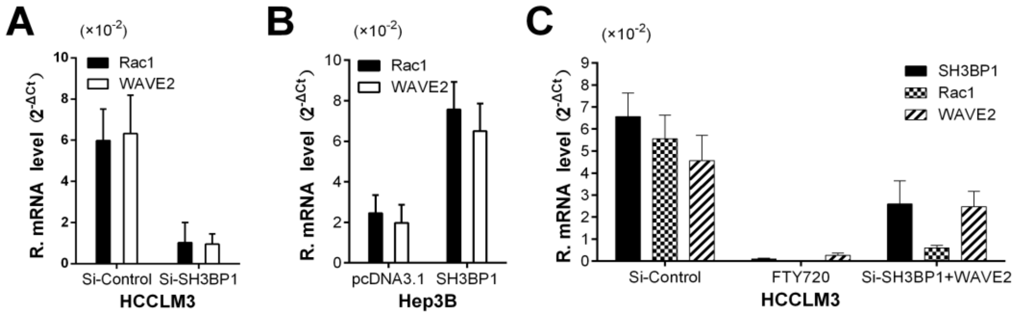

Figure S3: Results of mRMA expression was measured by qRT-PCR. (A) Expression of Rac1 and WAVE2 mRNA in infected Si-SH3BP1 HCCLM3 cells compare with Si-control. (B) Expression of Rac1 and WAVE2 mRNA in infected pcDNA3.1-SH3BP1 Hep3B cells compare with control group Hep3B cells. (C) The level of SH3BP1, WAVE2 and Rac1 mRNA in infected Si-SH3BP1 HCCLM3 or suppressed alone by FTY720.

Supplementary Table S1: Baseline demographics and clinicopathological characteristics of HCC patients in three cohorts.

Clinical and pathological indexes		Cohort Ⅰ (78 cases)		Cohort Ⅱ (326 cases)		Cohort Ⅲ (112 cases)	
		n	%	n	%	n	%
Age, years[a]	≤60	50	64.1	189	57.98	62	55.36
	＞60	28	35.9	137	42.02	50	44.64
Sex	Male	67	85.9	289	88.65	104	92.86
	Female	11	14.1	37	11.35	8	7.14
HBsAg	Negative	6	7.69	47	14.42	15	13.39
	positive	72	92.31	279	85.58	97	86.61
Albumin	＞35 g/L	69	88.46	295	90.5	98	87.5
	≤35 g/L	9	11.54	31	9.5	14	12.5
Child-Pugh classification	A	72	92.31	322	98.77	107	95.54
	B	6	7.69	4	2.23	5	4.46
AFP (ng/ml)	≤20	22	28.2	167	51.23	43	38.49
	＞20	56	71.8	159	48.77	69	61.61
Liver cirrhosis	Absent	10	12.82	31	9.51	9	8.04
	Present	68	87.18	295	90.49	103	91.96
Tumour encapsulation	Complete	32	41.03	138	42.33	49	43.75
	None	46	58.97	188	57.67	63	56.25
Tumour size (cm)	≤5	29	37.18	134	41.1	77	68.75
	＞5	49	62.82	192	58.9	35	31.25
Tumour number	Single	38	48.72	181	55.52	63	56.25
	Multiple*	40	51.28	145	44.48	49	43.75
Vascular invasion	Absent	45	57.69	272	84.44	57	50.89
	Present [§]	33	42.31	54	15.56	55	49.21
Edmondson-Steiner grade	Ⅰ-Ⅱ	52	66.67	253	77.6	72	64.29
	Ⅲ-Ⅳ	26	33.33	73	22.4	40	35.71
TNM stage	Ⅰ	40	51.28	153	46.9	72	64.28
	Ⅱ	23	29.49	148	45.4	38	33.93
	Ⅲ	15	19.23	25	7.7	2	1.79

Abbreviations: HCC, hepatocellular carcinoma; HBsAg, hepatitis B surface antigen; AFP, a-fetoprotein; TNM, tumour-node-metastasis

*Multiple was defined as: tumor number＞2.

§Vascular invasion (VI) present was defined as: gross invasion and microscopic invasion involvement of portal vein, hepatic vein, inferior vena cava (IVC).

Supplementary Table S2: Oligonucleotide sequences of specific primers for quantitative real-time PCR.

Gene symbol	Forward sequence (5’-3’)	Reverse sequence (5’-3’)
SH3BP1	TAGTCACAGCCATACGACCAT	TGCATTGCTCCACTTTCCTCT
Rac1	ATGTCCGTGCAAAGTGGTATC	CTCGGATCGCTTCGTCAAACA
WAVE2	AGCCTTCAGAAGTTCCACCA	CTGCAGCATCTTCTCCTTCC
VEGF	TCCAAGGATATTTCAGATTCTATA	ACAGTTGGTTGGGAAGTGAT
HIF-1α	CCAGTTAGGTTCCTTCGATCAGT	TTTGAGGACTTGCGC TTTCA
GAPDH	TGTCTGGCACATTGGACATT	GCACCGTCAAGGCTGAGAAC
β-actin	TGGTGAAGACGCCAGTGG A	TTGTTACAGGAAGTCCCTTGCC

All sequences of the primers for SYBR Green Ⅰ real-time PCR were obtained from on-line PrimerBank database (http://pga.mgh.harvard.edu/primerbank/), and were synthesized by TaKaRa, Dalian, China. Primers were designed to generate a PCR amplification product of 100 to 250 bp. Melting curve analysis was performed to assure that only one PCR product was formed. Only primer pairs yielding unique amplification products without primer dimer formation were subsequently used for real-time PCR assays.

Supplementary Table S3: The types, dilutions and sources of the primary antibodies used for Western blot and immunohistochemical analysis.

Antibody	Dilution WB	Dilution IHC	Species	Source -Cat. Number
SH3BP1	1:1000	1:1000	Rabbit polyclonal	Santa Cruze (cat. No.sc-86268)
Rac1	1:1000	1:1000	Mouse monoclonal	EMD Millipore (cat. No. 05-389)
WAVE2	1:500	1:1000	Mouse monoclonal	Cell Signaling Technology (cat. No. 3659S)
VEGF	1:2000	1:1000	Mouse monoclonal	EMD Millipore (cat. No. GF25-100UG)
HIF-1α	1:1000	1:2000	Mouse monoclonal	Thermo Scientific Pierce (cat. No. MA1-12612)
CD105	—	1:1000	Mouse monoclonal	EMD Millipore (cat. No. 05-1424)
β-actin	1:3000	—	Mouse monoclonal	Sigma-Aldrich (cat. No. A5441)

Supplementary Table S4: Oligonucleotide sequences of shRNA inserts (cloned into *Age*I and *Ecor*I sites of pLKO.1 lentiviral vector).

Sequences-1 targeting SH3BP1:

Sense:5'-*CCGGC* **CAGTCTCTTTGAGTAACCCT** *CTCGAG* **AGGGTTACTCAAAGAGACTGG** *TTTTTG*-3';Antisense:5'-AATTC**AAAAACCAGTCTCTTTGAGTA** *ACCCTC* **TCGAGAGGGTTACTCAAAGA** *GACTGG*-3'

Sequences-2 targeting SH3BP1:

Sense:5'-*CCGG* **CCTGGAGATTCAGGCCGATTA** CTCGAG **TAATCGGCCTGAATCTCCAGG** *TTTTG*-3'

Antisense:5'-*AATT* **CAAAAACCTGGAGATTCAGGCC** GATTAC **TCGAGTAATCGGCCTGAATCTC** *CAGG*-3'

*. **Sequences-3 targeting SH3BP1:**

Sense:5'-*CCGG* **GTTACCAAGGAGGACTCCTAT** *CTCGAG* **ATAGGAGTCCTCCTTGGTAAC** *TTTTTG*-3'

Antisense:5'-*AATT* **CAAAAAGTTACCAAGGAGGACT** CCTATC **TCGAGATAGGAGTCCTC** *CTTGGTAAC*-3'

§. **Control sequences of SH3BP1 shRNA:**

Sense:5'-*CCGG* **CATGGCAAACAAGGTGGAGAA** *CTCGAG* **TTCTCCACCTTGTTTGCCATG** *TTTTTG*-3'

Antisense:5'-*AATT* **CAAAAACATGGCAAACAAGGTGG** *AGAACT* **CGAGTTCTCCACCTTGTTT** *GCCATG*-3'

Sequences-1 targeting WAVE2:

Sense: 5'-*CCGG* ***GCC*** **ATATTCAACTACAGCCTT** *CTCGAG* **AAGGCTGTAGTTGAATATGGC** *TTTTTG*-3'

Antisense: 5'-*AATT* **CAAAAAGCCATATTCAACTA** *CAGCCT* **TCTCGAGAAGGCTGTAGTTGAA** *TATGGC*-3'

Sequences-2 targeting WAVE2:

Sense: 5'-*CCGGC* **AGTGGACAACCAAAGAGGAT** *CTCGA*G**ATCCTCTTTGGTTGTCCACTG** *TTTTTG*-3'

Antisense:5'-*AATTC* **AAAAACAGTGGACAACCAAAG** *AGGAT* **CTCGAGATCCTCTTTGGTTGT** *CCACTG*-3'

*. **Sequences-3 targeting WAVE2:**

Sense:5'-*CCGG* **CCTGTCTTAGAAACATACAAT** *CTCGAG* **ATTGTATGTTTCTAAGACAGG** *TTTTTG*-3'

Antisense: 5'-*AATT* **CAAAAACCTGTCTTAGAAACA** *TACAAT* **CTCGAGATTGTATGTTTCTAA** GACAGG-3'

§. **Control sequences of WAVE2 shRNA:**

Sense: 5'-*CCGG* **CCTTCCTTCTGGAACTCAGTT** *CTCGAG* **AACTGAGTTCCAGAAGGAAGG** *TTTTTG*-3'

Antisense: 5'-*AATT* **CAAAAACCTTCCTTCTGGAAC** *TCAGTT* **CTCGAGAACTGAGTTCCAGAA** *GGAAGG*-3'

*. Down-regulation efficiency of SH3BP1 and WAVE2 expression (>80%) was respectively confirmed using qRT-PCR and Western blot analysis.

§. Random sequences without any known gene targeting were respectively applied as the control inserts of SH3BP1 shRNA and WAVE2 shRNA sequences.

#. The titer of Si-SH3BP1, Si-WAVE2 and Si-Control lentivirus respectively was 1×10^9, 1×10^9 and 2×10^9 TU/mL. The optimal multiplicity of infection (MOI) for lentivirus transfection into HCC cells was 25 TU/mL.
